# A Rapidly Progressing Lower Extremity Soft Tissue Sarcoma in an Adolescent Patient

**DOI:** 10.7759/cureus.102963

**Published:** 2026-02-04

**Authors:** Chenxi Shi, Lydia Espinoza

**Affiliations:** 1 College of Osteopathic Medicine, Kansas City University of Medicine and Biosciences, Joplin, USA; 2 Internal Medicine, Mercy Hospital, Joplin, USA

**Keywords:** adolescent, lower extremity mass, metastatic sarcoma, sarcoma soft tissue, undifferentiated sarcoma

## Abstract

Soft tissue sarcomas (STSs) are rare malignant tumors of mesenchymal origin with heterogeneous histologic subtypes and variable clinical behavior. Although STSs most commonly arise in the extremities, rapidly progressive sarcomas involving the distal lower extremity in adolescents have not been well documented. We report a case of an STS in a previously healthy adolescent female whose initial presentation was presumed to be a muscle strain. Over a three-month period, her symptoms worsened with progressive enlargement of a calf mass and development of widespread metastatic disease. This case highlights the importance of maintaining suspicion for malignancy in rapidly enlarging soft tissue masses, with timely referral to specialized sarcoma centers for further evaluation.

## Introduction

Soft tissue sarcomas (STSs) are a rare type of malignant connective tissue tumor [1]. STSs can be found in various locations and are made up of different tissue subtypes. STSs most commonly arise in the extremities, accounting for approximately 60% of cases, with the lower extremity affected nearly three times more often than the upper extremity [2]. These tumors may also originate in the trunk, retroperitoneum, intra-abdominal cavity, and various other organs in the body [2]. In addition, STSs encompass a wide range of histologic subtypes and are classified based on their presumed tissue of origin; for example, malignant smooth muscle tumors are termed leiomyosarcomas. The fifth edition of the World Health Organization (WHO) Classification of Tumours recognizes more than 70 distinct subtypes of STSs based on histopathologic and molecular features [1]. Notably, the distribution of STS subtypes differs by age. In pediatric and adolescent populations, rhabdomyosarcoma accounts for about 50% of all STSs, whereas liposarcoma and leiomyosarcoma are some of the common entities in adults [3,4].

The majority of STS cases are idiopathic. Nonetheless, several hereditary cancer predisposition syndromes, including Li-Fraumeni syndrome, neurofibromatosis type I, and hereditary retinoblastoma, are associated with an increased risk of sarcoma development, contributing to the relatively higher prevalence observed in younger patients [5]. Prognosis and clinical behavior of STSs vary considerably by histologic subtype, tumor grade, size, and stage at diagnosis [6]. Growth rates range from indolent lesions that remain undetected for years to highly aggressive tumors with high metastatic potential. 

STSs also cause different burdens across age groups. The annual incidence is approximately 2-5 cases per 100,000 individuals, accounting for about 1% of all adult malignancies [7]. In contrast, STSs represent a larger proportion of cancers in the adolescent and young adult population, comprising approximately 7-10% of all malignancies in this age group [8]. Although it only accounts for a fraction of tumors, delayed diagnosis can result in substantial morbidity and poor clinical outcomes. In a prior analysis of 1,170 patients with STSs, distant metastases were present at diagnosis in approximately 10% of cases, with over 80% involving the lungs [9]. Patients with pulmonary metastases had a median survival of 11 months.

About 45% of all STSs arise from the thigh, while only about 10% of all STSs arise from the calf [10]. While previous case reports have described STS arising in the distal lower extremity, reports of rapidly progressive, aggressive STS involving the distal lower extremity in adolescents remain rare [11,12]. Here, we describe the clinical course and diagnostic evaluation of the rapid progression of an aggressive STS arising in the soleus muscle of an adolescent patient.

## Case presentation

A previously healthy female in her late teens with no significant past medical or family history presented to her primary care physician complaining of muscle ache in the right lower extremity that had been progressively worsening for one month. She denied a history of recent trauma. Physical examination was unremarkable except for increased muscle tightness in the right calf. There was no swelling, edema, or deformity. There was a negative Homan’s test bilaterally. Based on the initial presentation, the working diagnosis was a muscle strain, and symptomatic management was recommended. An ambulatory ultrasound was ordered, but was not obtained. The patient initially reported mild improvement in symptoms.

Given the patient’s age and clinical presentation, the initial differential diagnoses considered based on the order of epidemiologic likelihood include muscle strain, deep vein thrombosis, benign soft tissue mass, systemic lupus erythematosus (SLE), Lyme disease, rheumatoid arthritis, and malignancy.

Patient returned to the clinic two months later, complaining of worsening pain and enlargement of the calf mass. New associated symptoms included localized warmth and episodic severe pain. She additionally reported new pain in her shoulder and a single episode of possible clot in her urine. Physical examination revealed symmetric bilateral lower extremity on inspection; the right calf was firm and tender on palpation, and a palpable mass was present. Labs obtained during this visit showed microcytic anemia, normal iron study, normal urinalysis, positive antinuclear antibody (ANA) screen with all negative reflex autoimmune serologies, and elevated C-reactive protein (Table 1). 

**Table 1:** List of abnormal lab values Laboratory studies obtained three months after symptom onset demonstrated decreased hemoglobin with a normal MCV, consistent with normocytic anemia. An elevated C-reactive protein suggested underlying systemic inflammation. ANA screening was positive, with all reflex autoimmune testing negative. MCV, mean corpuscular volume; ANA, antinuclear antibody.

Test	Value
Hemoglobin	9.2 g/dL
MCV	81.5 fL
C-reactive protein	50.4 mg/L
ANA screen	Positive

The patient initially presented with muscle tightness and shoulder pain, raising concern for a systemic inflammatory process, including migratory polyarthritis and SLE. In the context of constipation, anemia of unclear etiology, and the absence of prior medical history in a young female patient, an autoimmune disorder was initially considered. However, the persistent enlargement of the lower extremity mass and its migratory and progressive symptoms prompted further diagnostic evaluation.

**Figure 1:**
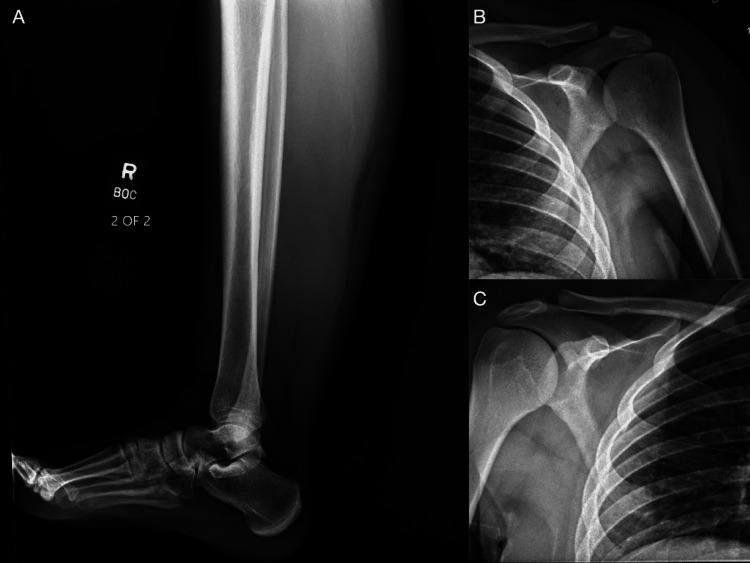
X-ray of tibia and fibula (A), bilateral shoulders (B and C) obtained approximately 3 months since symptom onset. All were unremarkable.

**Figure 2:**
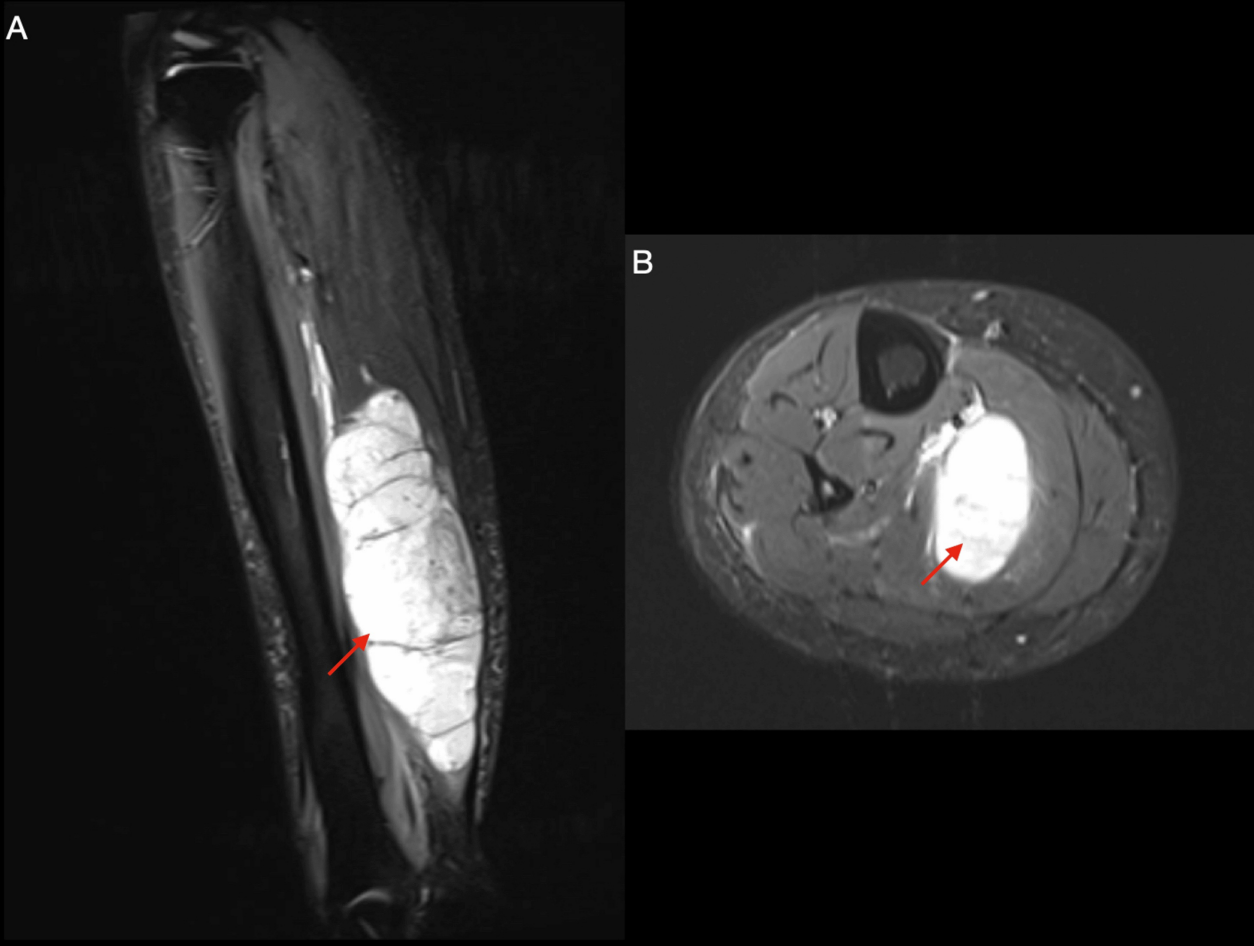
Sagittal (A) and axial (B) views of T2-weighted MRI of the right tibia and fibula, obtained 3.5 months after symptom onset, demonstrated a hyperintense intramuscular mass with internal septations (red arrows). Axial images showed intact gastrocnemius boundaries with a hyperintense lesion confined within the soleus muscle. The mass measures 182 x 51 x 54 mm in size.

**Figure 3:**
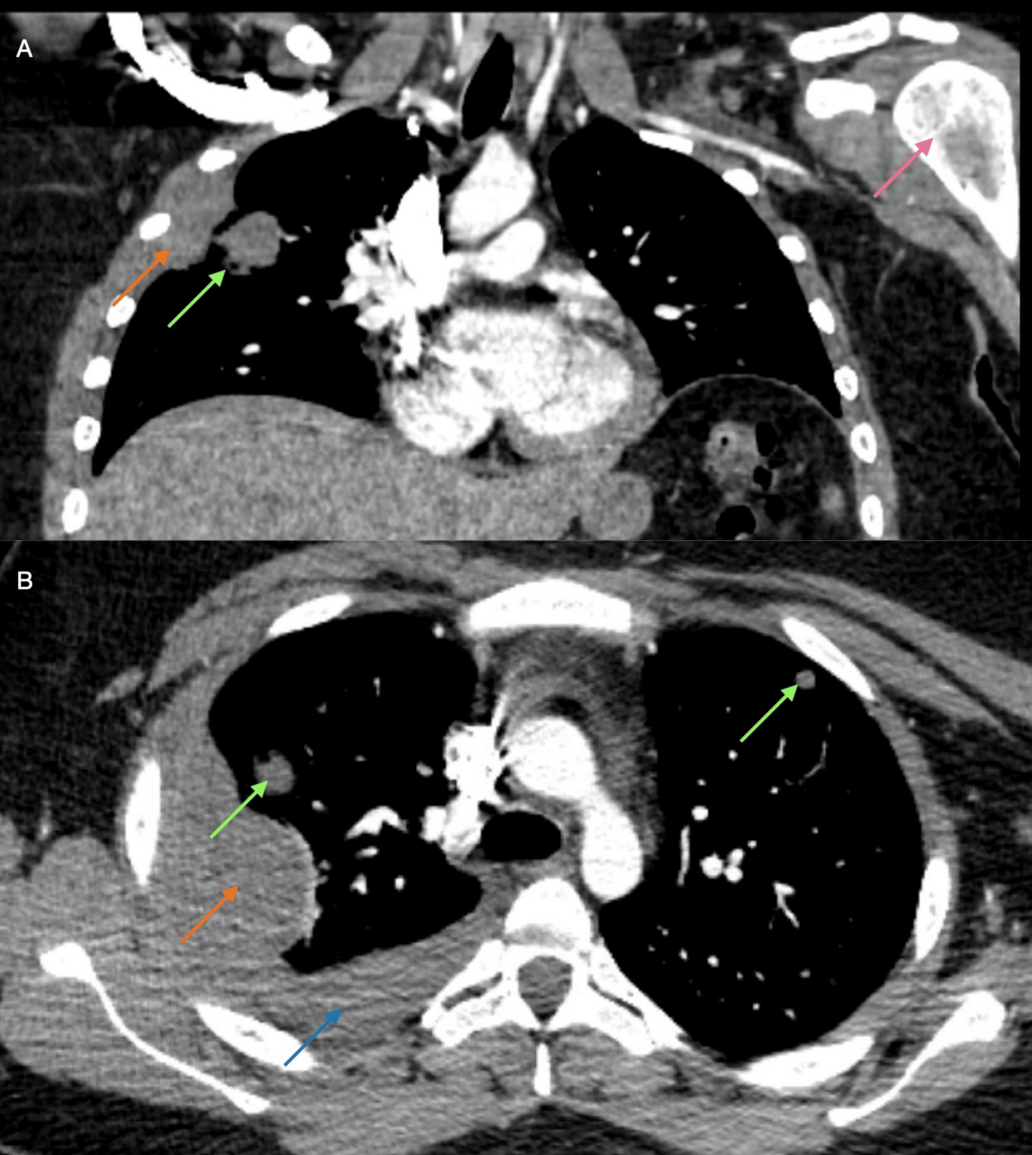
Selected coronal (A) and axial (B) views of CT chest with contrast obtained 3.5 months after symptom onset. Images show pulmonary metastases throughout both lungs (green arrows), pleural effusion (blue arrow), large pleural mass (orange arrows), and permeative appearance of the left proximal humerus (pink arrow). These findings are most consistent with metastatic disease.

Plain radiographs were obtained to evaluate both the shoulder pain and the right lower extremity mass. These studies were unrevealing; no calcifications or discrete osseous or soft tissue lesions were identified (Figure 1). Given the persistent concern, CT and MRI were subsequently performed. Lower extremity MRI revealed a large soft tissue mass within the right soleus muscle measuring approximately 18 × 5 9 × 5 cm (Figure 2). CT of the chest revealed multiple bilateral pulmonary nodules and pleural effusion consistent with metastatic disease, as well as osseous lesions involving the right lateral third rib and the left proximal humerus (Figure 3). Considering the size of the primary lesion and the presence of suspected metastatic disease, a malignant neoplastic process was strongly suspected. 

The patient was given an urgent referral to orthopedic oncology. Positron emission tomography (PET) and ultrasound-guided biopsy were performed. PET imaging demonstrated a markedly hypermetabolic mass in the right lower leg, with widespread metastatic involvement including the bilateral lungs, liver, and both axial and appendicular skeleton (images were unavailable due to testing performed at an outside facility). 

In the pathology report, tumor cells demonstrate a broad morphologic spectrum, ranging from small epithelioid to round and rhabdoid forms, with scattered markedly pleomorphic cells. Focal areas of myxoid stroma are present without definitive chondroid differentiation. Immunohistochemical studies show patchy CD99 positivity with retention of INI1 and SMARCA4 (BRG1) expression; tumor cells are negative for keratin AE1/AE3/PCK26, CD45, myogenin, desmin, SOX10, WT1 (nuclear), and S100 protein. Fluorescence in situ hybridization is negative for FOXO1 rearrangement, and sarcoma fusion panel testing (SARCP) is negative for gene fusions. Although precise classification remains indeterminate, the overall morphologic, immunophenotypic, and molecular findings argue against Ewing sarcoma and rhabdomyosarcoma (histology images were unavailable due to testing performed at an outside facility).

Surgical intervention was not recommended on the oncology team, given disseminated metastatic disease. The patient was initiated on a four-day AIM chemotherapy regimen consisting of doxorubicin (Adriamycin), ifosfamide, and mesna.

During the initial course of chemotherapy, treatment was complicated by respiratory distress secondary to pleural effusion requiring chest tube placement. Throughout her chemotherapy course, the patient also required multiple emergency department visits and hospitalizations for neutropenic fever treated with intravenous antibiotics and anemia requiring blood transfusions. Although the primary lower extremity lesion demonstrated interval improvement after cycle 2 of chemotherapy, the disease continued to progress with further metastatic spread, including the brain, despite aggressive treatment. The patient ultimately succumbed to her illness within six months of diagnosis.

## Discussion

STS can progress rapidly if not detected early, and effective treatment relies heavily on timely diagnosis. Delayed diagnosis often occurs because sarcomas may grow slowly or remain asymptomatic for years, or because benign soft tissue masses are more common, leading to low suspicion of malignancies [13,14]. Therefore, physicians should maintain a differential that includes sarcomas when evaluating large or fast-growing soft tissue masses. A thorough history and physical examination should be the foundation of all patient encounters. When sarcoma is suspected, initial workup should include cross-sectional imaging, such as CT or MRI, with prompt referral to a sarcoma specialist if imaging reveals concerning features.

Our patient presented with a rapidly progressing STS. Her initial localized complaint resembled a muscle strain, and in the absence of systemic symptoms such as weight loss and fatigue, sarcoma was initially considered unlikely. She was appropriately managed for a presumed soft tissue strain. However, due to persistent symptoms and repeated clinic visits, the diagnostic evaluation was appropriately escalated. A plain radiograph was unrevealing, which is not unexpected, given its limited sensitivity for soft tissue masses and the lack of bone invasions, although X-rays can sometimes identify calcifications or rule out bony involvement. Subsequent imaging revealed a large, heterogeneous intramuscular mass within the soleus muscle, along with multiple pulmonary lesions, highlighting the need for further evaluation. Definitive diagnosis of STS was made using tissue biopsy, with histologic and molecular analysis guiding classification. The single episode of a possible small blood clot observed in her urine was likely benign, given the absence of kidney or bladder invasion, but it may also have been related to malignancy-associated hypercoagulability.

PET scan obtained within four months of symptom onset demonstrated widespread systemic involvement, including the liver and lungs, which are not uncommon in aggressive sarcomas [15]. The rapid growth pattern in our patient raised concern for aggressive sarcomas, such as pleomorphic sarcoma or undifferentiated sarcoma [13,16]. Tissue biopsy revealed histologic and immunophenotypic features inconsistent with rhabdomyosarcoma or Ewing sarcoma, two common soft tissue tumors in our patient's age group [17]. Despite extensive molecular studies, the precise classification remained indeterminate. A recent study showed that about one-third of all sarcomas were not otherwise specified, though this could represent both truly undifferentiated tumors and cases where specific subtyping could not be obtained [18,19]. However, given the aggressive behavior and morphology, this could likely represent a case of undifferentiated STS. 

## Conclusions

This case underscores the potential for rapid progression in a subset of STSs and highlights several important clinical lessons. Early recognition, prompt imaging, tissue diagnosis, and referral to a multidisciplinary sarcoma center are essential for optimal management. While new diagnostic tools, such as next-generation sequencing, may aid in classification, there remains a critical need for further research into the clinical management and identification of these aggressive tumors.
